# Mechanical Stress as the Common Denominator between Chronic Inflammation, Cancer, and Alzheimer’s Disease

**DOI:** 10.3389/fonc.2015.00197

**Published:** 2015-09-17

**Authors:** Marcel Levy Nogueira, Jorgelindo da Veiga Moreira, Gian Franco Baronzio, Bruno Dubois, Jean-Marc Steyaert, Laurent Schwartz

**Affiliations:** ^1^Département de Neurologie, Institut de la Mémoire et de la Maladie d’Alzheimer (IM2A), Hôpital de la Pitié-Salpêtrière, AP-HP, Paris, France; ^2^Institut des Neurosciences Translationnelles de Paris (IHU-A-ICM), Institut du Cerveau et de la Moelle Epinière (ICM), Paris, France; ^3^UMR 7161, Laboratoire d’informatique (LIX), Ecole Polytechnique, Université Paris-Saclay, Palaiseau, France; ^4^Integrative Oncology, Kines Medical Center, Milan, Italy; ^5^UMR-S975, CNRS, INSERM, Institut du Cerveau et de la Moelle Epinière (ICM), Paris, France

**Keywords:** cancer, inflammation, fibrosis, pressure, Alzheimer’s disease, mechanical stress

## Abstract

The pathogenesis of common diseases, such as Alzheimer’s disease (AD) and cancer, are currently poorly understood. Inflammation is a common risk factor for cancer and AD. Recent data, provided by our group and from others, demonstrate that increased pressure and inflammation are synonymous. There is a continuous increase in pressure from inflammation to fibrosis and then cancer. This is in line with the numerous papers reporting high interstitial pressure in cancer. But most authors focus on the role of pressure in the lack of delivery of chemotherapy in the center of the tumor. Pressure may also be a key factor in carcinogenesis. Increased pressure is responsible for oncogene activation and cytokine secretion. Accumulation of mechanical stress plays a key role in the development of diseases of old age, such as cardiomyopathy, atherosclerosis, and osteoarthritis. Growing evidence suggest also a possible link between mechanical stress in the pathogenesis of AD. The aim of this review is to describe environmental and endogenous mechanical factors possibly playing a pivotal role in the mechanism of chronic inflammation, AD, and cancer.

## Introduction

Mechanical stress defines the distribution of forces exerted in a solid or fluid body being deformed as a result of external loads. Deformation changes the relative locations of molecules within a body which gives rise to internal forces balancing external loads. We already have an intuitive understanding of distribution of forces when we consider pressure. Mechanical stress is analogous to pressure when the forces are perpendicular to a surface (compressive or tensile stress). When the forces run parallel to a surface there is a shear stress. The influence of mechanical stress of living organisms is omnipresent. It depends not only on environmental and endogenous loads (pressure exerted by cavities and blood) but also on intrinsic mechanical factors of organs, such as shape, architecture, and mechanical properties of tissues (1, 2).

Mechanical stress could be the cause, the consequence, and/or might also simultaneously interact with biological processes. Cells are continuously subjected to mechanical forces that influence cell division, gene expression, cell migration, morphogenesis, cell adhesion, fluid homeostasis, ion channel gating, and vesicular transport (3–5). The seminal work of D’Arcy Thompson demonstrated that physical forces play a key role in plant and animal morphogenesis (6). Aging-associating diseases of different mechanisms, such as cardiomyopathy (7), degenerative valvular disease (8), atherosclerosis (9), and osteoarthritis (10), and cataract (11) present mechanical factors interacting with their pathogenesis. The aim of this review is to describe the potential role of mechanical stress as a pathway underlying the mechanism of chronic inflammation, cancer, and Alzheimer’s disease (AD). Cancer and AD are obviously different diseases but they share multiple common epidemiological and biological features.

## Common Denominators between Cancer and AD

In both cancer and AD, there are a small (<3%) but informative proportion of the patients who have inheritable genetically transmissible risk factors. More than 50 different genes are known to be involved in cancer. For example, BCRA1 and BCRA2 carriers are at a very risk not only for breast cancer but also for ovarian cancer and sarcoma. This risk is high enough (80%) as to warrant prophylactic surgery, such as mastectomies. To the difference with the much more common sporadic cancer, these tumors arise in young patients most commonly before the age of 50. Around 0.1% of the cases of AD are familial forms of autosomal-dominant inheritance, which usually have an onset before age 65 (12). Most of autosomal-dominant familial AD can be attributed to mutations in one of three genes: those encoding amyloid precursor protein (APP) and presenilin 1 and 2 (13). Most mutations in the APP and presenilin genes increase the production of a small protein called amyloid β (Aβ), which is the main component of senile plaques (14). Most cases of AD do not exhibit autosomal-dominant inheritance, and thus are termed sporadic AD. These genetic features are rare and should not let us miss the main features.

Cancer and AD share two major risk factors: age and sex. Two-thirds of cancer and AD arise after age 70 (15). Two-thirds of the patients are over 70 when diagnosed either with cancer or AD. The second risk factor is sex. Men are at a given age at a higher risk for cancer or AD. Moreover, aging is clearly linked to chronic inflammation; aging is associated with increased levels of chronic inflammation and with inflammatory activity reflected by increased circulating levels of cytokines, such as tumor necrosis factor (TNF) and interleukin (IL) 6, and acute phase proteins (16). Various inflammatory processes and cytokines may also have a role in the pathology of AD. Inflammation is a general marker of tissue damage in any disease, and may be either secondary to tissue damage in AD or a marker of an immunological response (17).

## Inflammation as a Common Risk Factor for AD and Cancer

Inflammation is a common risk factor for cancer and AD. Liver cancer is frequently associated with such pre-existing inflammation and fibrosis. Between 60 and 90% of hepatocellular carcinoma occur in patients with hepatic macronodular cirrhosis (18, 19). Chronic liver disease of any type is a risk factor for liver cancer. Evidence for a cause-effect link between cirrhosis and hepatocellular carcinoma is lacking. The relation may often be one of chance alone, since not all cirrhotics develop cancer. Nonetheless, diseases that cause cirrhosis also increase the risk of hepatocellular carcinoma (19). Furthermore, the more disorganized the liver becomes, the higher the risk of hepatocellular carcinoma (18, 19). The same is true for lung cancer, which is very often preceded by chronic bronchitis (inflammation of the main bronchi). Chronic bronchitis paves the way toward lung cancer. Long-term usage of non-steroidal anti-inflammatory drugs (NSAIDs) is associated with a reduced likelihood of developing AD (20) and cancer (21). In addition to long-standing evidence from observational studies, evidence from randomized trials of the effectiveness of aspirin for chemoprevention of colorectal cancer has increased substantially in recent years. Trials have shown that daily aspirin for about 5 years reduces incidence and mortality due to colorectal cancer by 30–40% after 20 years of follow-up, and reduces the 20-year risk of all-cause cancer mortality by about 20%. Human post-mortem studies, in animal models, or *in vitro* investigations also support the notion that NSAIDs can reduce inflammation related to amyloid plaques (20). However, trials investigating their use as palliative treatment have failed to show positive results while no prevention trial has been completed (20).

## Inflammation is Synonymous to Increased Osmotic Pressure

The word “physician” is a reminiscence of the time when medicine was a part of Physics. But today, the role of physical forces in the development of disease has been largely neglected (to the notable exception of orthopedics). Inflammation is characterized by *tumor*, *dolor*, *rubor*, and *calor* as stated by Galen 2000 years ago. Inflammation can be caused by factors as diverse as heat, freezing temperature, trauma, or chemicals. The capillary hydrostatic pressure drives fluid out of the capillary (i.e., filtration), and is highest at the arteriolar end of the capillary and lowest at the venular end. The oncotic pressure drives the liquid back into the capillary. Because the capillary barrier is readily permeable to ions, the osmotic pressure within the capillary is principally determined by plasma proteins that are relatively insoluble. Therefore, instead of speaking of “osmotic” pressure, this pressure is referred to as the “oncotic” pressure or “colloid osmotic” pressure because it is generated by colloids. Albumin generates about 70% of the oncotic pressure. This pressure is typically 25–30 mmHg.

During inflammation, there is extravasation of proteins from the vascular space to the extracellular space. It is known to the clinicians that there is an increase of protein content in the inflammatory fluid, such as a pleural effusion or a pericarditis. This increased protein content and the resulting increased osmotic pressure is the reason for inflammation. We have previously shown both *in vivo* and *in vitro* that hyperosmolarity can induce pro-inflammatory cytokine responses in epithelial cells (22–24). Our group and others recently demonstrated that inflammation results from increased interstitial pressure (16, 22–24). There is increased osmotic pressure in the inflammatory fluid due to the presence of a large quantity of proteins. This increased extracellular osmolarity results in the secretion of pro-inflammatory cytokines, such as TNF, IL 6 and 8, and numerous other cytokines. Monocytes exposed to hyperosmolarity doubled their half live (24). It is probable that inflammation (a disease) is synonymous to increased osmolarity pressure (a force).

## Mechanical Stress as a Critical Factor in Cancer

Many solid tumors show an increased interstitial fluid pressure (IFP), which forms a barrier to transcapillary transport. This barrier is an obstacle in tumor treatment, as it results in inefficient uptake of therapeutic agents. There are a number of factors that contribute to increase IFP in the tumor, such as vessel abnormalities, fibrosis, and contraction of the interstitial matrix. It is this increased pressure that the physician tries to feel when doing a clinical exam, such a rectal exam when searching for a prostate cancer.

The role of increased pressure in carcinogenesis has been poorly investigated. There is a continuum of increased pressure from normal tissue to inflammation fibrosis and cancer. The pressure of the hepatic parenchyma is 4 mmHg. The parenchyma is under increased pressure in liver cirrhosis (13 mmHg) and in primary or metastatic cancer (between 15 and 25 mmHg). The increased interstitial pressure may be responsible for the most common features of cirrhosis and/or cancer, such as hepatocyte necrosis, extensive fibrosis, connective tissue deposition, vascular distortion, infiltration by immune cells, and nodular regeneration of the remaining tissue parenchyma. Increased pressure is known to induce collagen deposition and modulate cell proliferation either by cell death or by cell multiplication (25). Exposure of immune cells to increased osmotic pressure doubled their half-life (23).

Increased pressure is responsible for cancer invasion. Cancer invades preferentially soft tissues such as glands or muscle rather than fascia or bone (15). It is also because of increased pressure that cells can escape, reach the blood vessels to form distant metastasis. Physical forces play also a major role in cell proliferation (25–27). Mechanical deformation induces cell proliferation (26). Cell proliferation of colon carcinoma cell line, HCT116, is increased by 30% after 2 days of deformation (30 cycles/min). But solid stress (45–120 mmHg) inhibits multicellular tumor spheroid (27). Changes in physical constraints has another consequence (28), the probability of a wrong plane of cell division is increased. Transition from normal, well stratified epithelium, to an invasive, fractal, dendritic pattern is observed. This transition shows a sequence of morphologies in the following order as a function of loss of polarity: first, an apparently normal but already diseased tissue, then, metaplasic followed by a dysplasic tissue, and eventually carcinoma first *in situ*, then invasive. In fact, most normal cells, and especially the epithelial cells are organized along a structural plane (29), which allows cell adhesion to the mesenchyme on one side, and epithelium function on the lumen side.

Changes in physical constraints explain the stellar dendritic shape of cancer, enabling the cells to escape physical constraints from their neighbors (25, 28). This functional polarity is most often lost during carcinogenesis (29). In normal tissue, it is maintained by the cytoskeleton, by vesicular trafficking that proceeds along the microtubules (30), by organelles, such as the centrosomes, and in general terms, by the interpretation of cues coming from the surrounding tissue. In particular, mechanical forces coming from neighboring cells are able to induce a polarity of the cell, and influence cell divisions in specific directions. This question has already been studied in plants, where it is known that one can induce deterministically the position of the plane of division by a mechanical stress (31).

## Interactions between Mechanical Stress and AD

### Mechanical stress may influence AD pathophysiology

Several authors have already suggested the hypothesis that AD pathology is driven by mechanical forces. Wostyn et al. and Silverberg et al. were the first to bring up the causative link between intracranial pressure and AD (32–36). It has been shown that mechanical impedance (a measure of how much a structure resists motion when subjected to a given force) of the intracranial cavity and vessels plays a role in the pathophysiology of AD (37). Some have set forth that the strength of the pulse waves induced by the vascular tree in the craniospinal cavity is the underlying vascular pathophysiology behind AD and other conditions, such as vascular dementia and normal pressure hydrocephalus (NPH) (38–41). Barz (42) set forth that mechanical changes accumulate in neuronal membranes and cytoplasm in old age, in a similar fashion to how vessel walls stiffen and change in arteriosclerosis. Hachiya et al. (43) suggested that continuous and repetitive exposure to environmental mechanical stress, mostly in an unrecognized and inevitable manner in daily life, becomes a potential driving force for Aβ and tau aggregation (43).

A great number of studies have suggested that amyloid cascade may occur in parallel and their onset and rate could be under the influence of a series of environmental risk factors (44). Mechanical stress has been positioned as an environmental factor that may provoke disturbances in the cellular quality control systems and molecular chaperones that target misfolded proteins, such as tau and Aβ (43). Computational simulations have shown that Aβ structure could be twisted, flexed, and bent by the imposition of shear forces (45), which suggests that shear stress may influence Aβ misfolding and aggregation. Tau proteins, in turn, may resist and accumulate elastic stress, which seems to protect axonal microtubule from mechanical deformation (46). Amyloid peptides, others than Aβ, have been described in mechanical loading environments, such as heart valves exposed to high shear stress (47) or in the joint cartilage (48). Amyloid adhesion and nucleation, critical events in Aβ aggregation, can be mechanically induced by tensional forces exerted in amyloid nanofibers (49, 50). Atomistic simulations also showed that helical nanostructure of Aβ oligomers may induce neurotoxicity by mechanical damage on membrane structure (51).

### AD risk factors may be linked to mechanical stress exposure

Epidemiological and neuropathological data suggest a tight association between AD and the exposure to mechanical stress factors. Extracranial mechanical stressors predisposes an individual to AD later in life, as observed in traumatic brain injury (TBI) (52) and occupational exposure in athletes (boxers, football, and soccer players) and military personnel (53). Neuropathological analysis of TBI tissues in humans has led to notable findings regarding AD pathological features. Abnormal accumulation of Aβ deposits (54) and cytoskeletal tau proteins, pathological hallmarks of AD, has been constantly detected after isolated or/and repetitive cranial impacts (55, 56).

Mechanical energy can also exert stress within intracranial environment. Cerebrovascular blood flow accompanying every heartbeat generates forces that can displace the brain tissue by tens of micrometers (57). Similarly, mechanical interactions constantly occur between brain parenchyma and cerebrospinal fluid (CSF) (58). These mechanical stressors interacting with brain compartments are confined within the rigid structure of the skull. Changes in intracranial hydrodynamics may result in an increased risk of AD as seen in NPH, since several studies have demonstrated neuropathological evidence of AD in NPH patients (59–62). NPH is characterized by transient intracranial pressure peaks, while CSF pressure measurements usually remain within the normal range. The accumulation of hydrodynamic loads leads to chronic mechanical stress on the ventricular walls, and ultimately on the brain parenchyma (63). The frequency of tau-amyloid deposits through cortical biopsies taken during shunt placement has been shown to be greater than that of the general population, suggesting that NPH and AD may occur as a result a coexisting hydrodynamic pathophysiology (64–66).

Cerebrovascular hemodynamic stress, caused by atherosclerosis, heart diseases, and arterial hypertension, also affects cognition and are among the most important risk factors for AD (67). These vascular conditions interact with intracranial mechanical constraints, and so could be considered as “mechanical” risk factors (68). Arterial hypertension increases brain hemodynamic stress as a result of pulsating shock waves (some 30 million/year) produced by the external surface of the arterial wall in contact with the brain parenchyma (69). Atherosclerosis can increase brain arterial stiffness, increasing mechanical damage of the perivascular tissue (70) and leading to accumulation of Aβ plaques (71) and cognitive decline (40, 72).

### Mechanical stress models of AD-like pathology

Effects related to endogenous mechanical energy in AD pathology have been widely overlooked in hypotheses involving a postulated molecular amyloido-centric pathway, such as the amyloid cascade theory (73) derived from reductionistic transgenic animal mutation models that do not account for the principles of mechanics. Extracellular accumulation of Aβ and intracellular accumulation of tau in brain tissues and neuroinflammation have been described not only in transgenic animal models of AD but also in mechanical stress-based diseases of different mechanisms, such as TBI, arterial hypertension, and NPH. As observed in humans, AD-like pathology is also present in numerous experimental models of TBI using mice, rats, rabbits, pigs, and monkeys (74). Animal models of NPH have revealed accumulation of tau and Aβ deposits in brain tissues subjected to hydrodynamic stress (75, 76). It have been also found that rodent models subject to high levels of blood pressure, or hemodynamic stress, demonstrate an accumulation of Aβ and tau aggregates (77, 78). These mechanical stress models may increase our knowledge of how different mechanical factors, from both the external and the internal environment, could influence pathophysiological mechanisms underlying AD. Table 1 compares five hallmark features from transgenic and mechanical stress rodent models of AD pathology. Amyloidosis, tauopathy, gliosis, neuronal loss, or cognitive impairment could be present in at least four of the five selected mechanisms of mechanical stress.

**Table 1:** **Selected transgenic and mechanical stress-based models displaying AD pathological hallmarks**.

Rodent models	Transgenic			Mechanical stress			Arterial hypertension	HPN
		
	APP	Tau	APPxTau	Traumatic brain injury				
				CCI	FPI	Mld/WD
Amyloidosis	++	−	+	++	+	++	++	+
Tauopathy	?^a^	++	+	++	+	?^b^	+	+
Neuroinflammation	++	+	+	++	+	+	++	+
Neuronal loss	?^c^	?^c^	−	++	+	++	?^d^	+
Cognitive impairment	++	++	+	++	++	++	?^d^	+
Reference	(79–81)		(79–82)	(83–91)	(92–97)	(83, 90, 98, 99)	(77, 78, 100)	(75–106)

*Comparison between transgenic and mechanical stress rodent models as a function of five hallmark features of AD pathology*.

*(+) Feature present in mice or rat models*.

*(++) Feature present in both mice and rat models*.

*(?) Unknow*.

*Only increased p-tau but no tau deposits*.

*Tau transgenic mouse*.

*Only present in a few models*.

*Only when associated with white matter and focal lesions*.

*APP, amyloid precursor protein; CCI, controlled cortical impact; FPI, fluid percussion injury; Mld, mild repetitive trauma; WD, weight drop; NPH, normal pressure hydrocephalus*.

### Mechanics related to brain atrophy and brain amyloidosis in AD

In general, biological tissues adapt to higher levels of stress by changing cross-sectional area, density, or volume. In a recent work, our group estimated increments of mechanical stress undergoes by the tissues during brain shrinkage. Selecting AD as a primary model of a non-linear dynamic, chronically progressive degenerative disease, pressure equivalents were related to atrophy and found to be 42% higher in AD brains comparatively to normal aging brains (5.92 versus 3.43 mmHg, respectively). The phenomenon of mechanical brain fatigue, or the increased amplitude of oscillations generated by arterial, hydraulic, or external shock waves, was suggested to mainly contribute to the accumulation of mechanical stress in AD (107). Another example of how organs adapt to stress consists in changes in its mechanical properties. In AD, slow cumulative changes in the microarchitecture of the brain could impact its mechanical properties (108, 109). The elasticity and stiffness of the brain vary substantially in normal humans as well as with age and the state of the disease (110). Cerebral stiffness, which can be measured by magnetic resonance elastography (MRE) (111), decreases when AD pathology is present in both humans and transgenic mice models (108, 112). However, the extent to which mechanical dynamics influence AD pathophysiology, and vice-versa, remains a mystery. AD deposits could increase the resilience of neuronal tissues to mechanical stressors and consequently increase the tolerance to subsequent stresses. Like muscle fibers, neuronal tissues may adapt to physical stresses by altering their structure and composition to better meet the biological requirements of routine energy loads. It raises the possibility that mechanical stress levels, which exceed the maintenance range of brain tissues, could trigger and accelerate protein misfolding, aggregation, and deposition. The higher the exposure to mechanical stress, the more likely an early onset of the disease will be (43).

## Conclusion

The role of mechanical forces in the pathogenesis of chronic inflammation, AD, and cancer has been overlooked. In addition to genetic factors, the accumulation of mechanical energy could underlie biological cascade pathways in inflammation, cancer, and AD (Table 2). This hypothesis has to be tested before any conclusion can be drawn. The extent to which mechanical energy influences the pathophysiology of these conditions, and/or whether mechanics is just an effect of biological processes, remains a mystery. Mechanical stress could be the cause, the consequence, and/or might also simultaneously interact with biological processes. We point to the importance of accumulated mechanical stress as an environmental and endogenous factor that pushes cell and tissue functioning over the disease threshold.

**Table 2:** **Core hypotheses liking mechanical stress and the pathophysiology of cancer, inflammation, and Alzheimer’s disease (AD)**.

Condition	Hypothesis
Cancer	Increased interstitial pressure may accelerate carcinogenesis
	Mechanical forces may induce cell polarity, and consequently modulate cell divisions in specific directions
Chronic inflammation	Increased interstitial pressure (hyperosmolarity) may drive inflammation
AD	Accumulation of mechanical energy during lifespan may accelerate amyloid cascade, tauopathy, and microarchitectural changings in brain tissues

## Author Contributions

All the authors contributed equally to this manuscript.

## Conflict of Interest Statement

The authors declare that the research was conducted in the absence of any commercial or financial relationships that could be construed as a potential conflict of interest.

## References

[1] Jacobs CR Huang H Kwon RY Introduction to Cell Mechanics and Mechanobiology. New York, NY: Garland Science (2012).

[2] Brinckmann P Frobin W Leivseth G Musculoskeletal Biomechanics. Stuttgart. New York, NY: Thieme Medical Publishers Inc (2013).

[3] Hamill OP Martinac B. Molecular basis of mechanotransduction in living cells. Physiol Rev (2001) 81(2):685–740. 11274342 10.1152/physrev.2001.81.2.685

[4] Kim D-H Wong PK Park J Levchenko A Sun Y. Microengineered platforms for cell mechanobiology. Annu Rev Biomed Eng (2009) 11:203–33. 10.1146/annurev-bioeng-061008-124915 19400708

[5] Eyckmans J Boudou T Yu X Chen CS. A hitchhiker’s guide to mechanobiology. Dev Cell (2011) 21(1):35–47. 10.1016/j.devcel.2011.06.015 21763607 PMC3155761

[6] Thompson DW The forms of cells. In: Bonner JT, editor. On Growth and Form. Cambridge: Cambridge University Press (1942). 368 p.

[7] Modesto K Sengupta PP. Myocardial mechanics in cardiomyopathies. Prog Cardiovasc Dis (2014) 57(1):111–24. 10.1016/j.pcad.2014.03.003 25081406

[8] Robicsek F Thubrikar MJ. Mechanical stress as cause of aortic valve disease. Presentation of a new aortic root prosthesis. Acta Chir Belg (2002) 102(1):1–6. 11925731 10.1080/00015458.2002.11679253

[9] Heo K-S Fujiwara K Abe J. Shear stress and atherosclerosis. Mol Cells (2014) 37(6):435–40. 10.14348/molcells.2014.0078 24781409 PMC4086336

[10] Visser AW de Mutsert R le Cessie S den Heijer M Rosendaal FR Kloppenburg M The relative contribution of mechanical stress and systemic processes in different types of osteoarthritis: the NEO study. Ann Rheum Dis (2014). 10.1136/annrheumdis-2013-205012 24845389

[11] Michael R Barraquer RI Willekens B van Marle J Vrensen GFJM. Morphology of age-related cuneiform cortical cataracts: the case for mechanical stress. Vision Res (2008) 48(4):626–34. 10.1016/j.visres.2007.12.005 18221767

[12] Blennow K de Leon MJ Zetterberg H. Alzheimer’s disease. Lancet (2006) 368(9533):387–403. 10.1016/S0140-6736(06)69113-7 16876668

[13] Waring SC Rosenberg RN. Genome-wide association studies in Alzheimer disease. Arch Neurol (2008) 65(3):329–34. 10.1001/archneur.65.3.329 18332245

[14] Selkoe DJ. Translating cell biology into therapeutic advances in Alzheimer’s disease. Nature (1999) 399(6738 Suppl):A23–31. 10.1038/19866 10392577

[15] Schwartz L Cancer – Between Glycolysis and Physical Constraint: Between Glycolysis and Physical Constraint. Berlin Heidelberg: Springer Science & Business Media (2004). 172 p.

[16] Grimble RF. Inflammatory response in the elderly. Curr Opin Clin Nutr Metab Care (2003) 6(1):21–9. 10.1097/00075197-200301000-00005 12496677

[17] Greig NH Mattson MP Perry T Chan SL Giordano T Sambamurti K New therapeutic strategies and drug candidates for neurodegenerative diseases: p53 and TNF-alpha inhibitors, and GLP-1 receptor agonists. Ann N Y Acad Sci (2004) 1035:290–315. 10.1196/annals.1332.018 15681814

[18] DeVita V Lawrence T Rosenberg S DePinho R Weinberg R Cancer of the liver. In: DeVita Hellman editors. Rosenberg’s Cancer: Principles and Practice of Oncology. 9th ed North American Edition. Philadelphia, PA: LWW (2011). 2800 p.

[19] Podolsky DIK Alcohol-Related Disease and Cirrhosis. Harrison’s Principles of Internal Medicine. 13th ed New York: McGraw Hill (1994). p. 1483–95.

[20] Szekely CA Town T Zandi PP. NSAIDs for the chemoprevention of Alzheimer’s disease. Subcell Biochem (2007) 42:229–48. 10.1007/1-4020-5688-5_11 17612054

[21] Rothwell PM. Aspirin in prevention of sporadic colorectal cancer: current clinical evidence and overall balance of risks and benefits. Recent Results Cancer Res (2013) 191:121–42. 10.1007/978-3-642-30331-9_7 22893203

[22] Schwartz L Guais A Pooya M Abolhassani M. Is inflammation a consequence of extracellular hyperosmolarity? J Inflamm (Lond) (2009) 6:21. 10.1186/1476-9255-6-21 19549308 PMC2709204

[23] Abolhassani M Wertz X Pooya M Chaumet-Riffaud P Guais A Schwartz L. Hyperosmolarity causes inflammation through the methylation of protein phosphatase 2A. Inflamm Res (2008) 57(9):419–29. 10.1007/s00011-007-7213-0 18777115

[24] Schwartz L Abolhassani M Pooya M Steyaert J-M Wertz X Israël M Hyperosmotic stress contributes to mouse colonic inflammation through the methylation of protein phosphatase 2A. Am J Physiol Gastrointest Liver Physiol (2008) 295(5):G934–41. 10.1152/ajpgi.90296.2008 18755808

[25] Schwartz L Balosso J Baillet F Brun B Amman JP Sasco AJ. Cancer: the role of extracellular disease. Med Hypotheses (2002) 58(4):340–6. 10.1054/mehy.2001.1539 12027530

[26] Johnson FE Zhou M Collins BT Huang JS. Mechanical deformation induces proliferation of human colorectal carcinoma cells. Int J Oncol (2000) 16(3):617–22. 10675497 10.3892/ijo.16.3.617

[27] Helmlinger G Netti PA Lichtenbeld HC Melder RJ Jain RK. Solid stress inhibits the growth of multicellular tumor spheroids. Nat Biotechnol (1997) 15(8):778–83. 10.1038/nbt0897-778 9255794

[28] Fleury V Schwartz L Numerical investigation of the effect of loss of cellular polarity on cancer invasiveness and geometry. Fractals (2003) 11(04):397–414. 10.1142/S0218348X0300204X

[29] Locke D. Gap junctions in normal and neoplastic mammary gland. J Pathol (1998) 186(4):343–9. 10.1002/(SICI)1096-9896(199812)186:4<343::AID-PATH189>3.3.CO;2-O 10209481

[30] Lingle WL Lutz WH Ingle JN Maihle NJ Salisbury JL. Centrosome hypertrophy in human breast tumors: implications for genomic stability and cell polarity. Proc Natl Acad Sci U S A (1998) 95(6):2950–5. 10.1073/pnas.95.6.2950 9501196 PMC19675

[31] Peters WS Tomos AD The history of tissue tension. Ann Bot (1996) 77(6):657–65. 10.1093/aob/77.6.657 11541099

[32] Silverberg G Mayo M Saul T Fellmann J McGuire D. Elevated cerebrospinal fluid pressure in patients with Alzheimer’s disease. Cerebrospinal Fluid Res (2006) 3:7. 10.1186/1743-8454-3-7 16737542 PMC1538629

[33] Wostyn P. Intracranial pressure and Alzheimer’s disease: a hypothesis. Med Hypotheses (1994) 43(4):219–22. 10.1016/0306-9877(94)90069-8 7838004

[34] Wostyn P. Can chronic increased intracranial pressure or exposure to repetitive intermittent intracranial pressure elevations raise your risk for Alzheimer’s disease? Med Hypotheses (2004) 62(6):925–30. 10.1016/j.mehy.2004.01.013 15142650

[35] Wostyn P Audenaert K De Deyn PP. Alzheimer’s disease-related changes in diseases characterized by elevation of intracranial or intraocular pressure. Clin Neurol Neurosurg (2008) 110(2):101–9. 10.1016/j.clineuro.2007.10.011 18061341

[36] Wostyn P Audenaert K De Deyn PP. Alzheimer’s disease: cerebral glaucoma? Med Hypotheses (2010) 74(6):973–7. 10.1016/j.mehy.2009.12.019 20056337

[37] Bateman GA. The role of altered impedance in the pathophysiology of normal pressure hydrocephalus, Alzheimer’s disease and syringomyelia. Med Hypotheses (2004) 63(6):980–5. 10.1016/j.mehy.2004.04.019 15504565

[38] El Sankari S Gondry-Jouet C Fichten A Godefroy O Serot JM Deramond H Cerebrospinal fluid and blood flow in mild cognitive impairment and Alzheimer’s disease: a differential diagnosis from idiopathic normal pressure hydrocephalus. Fluids Barriers CNS. (2011) 8(1):12. 10.1186/2045-8118-8-12 21349149 PMC3045982

[39] Bateman GA. Pulse wave encephalopathy: a spectrum hypothesis incorporating Alzheimer’s disease, vascular dementia and normal pressure hydrocephalus. Med Hypotheses (2004) 62(2):182–7. 10.1016/S0306-9877(03)00330-X 14962623

[40] Bateman GA Levi CR Schofield P Wang Y Lovett EC. The venous manifestations of pulse wave encephalopathy: windkessel dysfunction in normal aging and senile dementia. Neuroradiology (2008) 50(6):491–7. 10.1007/s00234-008-0374-x 18379767

[41] Nagai K Akishita M Machida A Sonohara K Ohni M Toba K Correlation between pulse wave velocity and cognitive function in nonvascular dementia. J Am Geriatr Soc (2004) 52(6):1037–8. 10.1111/j.1532-5415.2004.52277_15.x 15161489

[42] Barz H Schreiber A Barz U. Impulses and pressure waves cause excitement and conduction in the nervous system. Med Hypotheses (2013) 81(5):768–72. 10.1016/j.mehy.2013.07.049 23953966

[43] Hachiya NS Kozuka Y Kaneko K. Mechanical stress and formation of protein aggregates in neurodegenerative disorders. Med Hypotheses (2008) 70(5):1034–7. 10.1016/j.mehy.2007.06.043 17910993

[44] Chetelat G. Alzheimer disease: aβ-independent processes-rethinking preclinical AD. Nat Rev Neurol (2013) 9(3):123–4. 10.1038/nrneurol.2013.21 23399647 PMC3935395

[45] Xu Z Paparcone R Buehler MJ. Alzheimer’s abeta(1-40) amyloid fibrils feature size-dependent mechanical properties. Biophys J (2010) 98(10):2053–62. 10.1016/j.bpj.2009.12.4317 20483312 PMC2872369

[46] Lazarus C Soheilypour M Mofrad MRK. Torsional behavior of axonal microtubule bundles. Biophys J (2015) 109(2):231–9. 10.1016/j.bpj.2015.06.029 26200859 PMC4621814

[47] Kristen AV Schnabel PA Winter B Helmke BM Longerich T Hardt S High prevalence of amyloid in 150 surgically removed heart valves—a comparison of histological and clinical data reveals a correlation to atheroinflammatory conditions. Cardiovasc Pathol (2010) 19(4):228–35. 10.1016/j.carpath.2009.04.005 19502085

[48] Niggemeyer O Steinhagen J Fuerst M Zustin J Rüther W. Amyloid deposition in rheumatoid arthritis of the hip. Rheumatol Int (2012) 32(9):2645–51. 10.1007/s00296-011-2005-9 21786121

[49] Varongchayakul N Johnson S Quabili T Cappello J Ghandehari H Solares SDJ Direct observation of amyloid nucleation under nanomechanical stretching. ACS Nano (2013) 7(9):7734–43. 10.1021/nn402322k 23987654

[50] Solar M Buehler MJ. Deformation behavior and mechanical properties of amyloid protein nanowires. J Mech Behav Biomed Mater (2013) 19:43–9. 10.1016/j.jmbbm.2012.10.007 23290516

[51] Pannuzzo M Milardi D Raudino A Karttunen M La Rosa C. Analytical model and multiscale simulations of Aβ peptide aggregation in lipid membranes: towards a unifying description of conformational transitions, oligomerization and membrane damage. Phys Chem Chem Phys (2013) 15(23):8940–51. 10.1039/c3cp44539a 23588697

[52] Chauhan NB. Chronic neurodegenerative consequences of traumatic brain injury. Restor Neurol Neurosci (2014) 32(2):337–65. 10.3233/RNN-130354 24398724

[53] Stein TD Alvarez VE McKee AC. Chronic traumatic encephalopathy: a spectrum of neuropathological changes following repetitive brain trauma in athletes and military personnel. Alzheimers Res Ther (2014) 6(1):4. 10.1186/alzrt234 24423082 PMC3979082

[54] Gatson JW Warren V Abdelfattah K Wolf S Hynan LS Moore C Detection of β-amyloid oligomers as a predictor of neurological outcome after brain injury. J Neurosurg (2013) 118(6):1336–42. 10.3171/2013.2.JNS121771 23540266

[55] Kanayama G Takeda M Niigawa H Ikura Y Tamii H Taniguchi N The effects of repetitive mild brain injury on cytoskeletal protein and behavior. Methods Find Exp Clin Pharmacol (1996) 18(2):105–15. 8740242

[56] Kane MJ Angoa-Pérez M Briggs DI Viano DC Kreipke CW Kuhn DM. A mouse model of human repetitive mild traumatic brain injury. J Neurosci Methods (2012) 203(1):41–9. 10.1016/j.jneumeth.2011.09.003 21930157 PMC3221913

[57] Kucewicz JC Dunmire B Leotta DF Panagiotides H Paun M Beach KW. Functional tissue pulsatility imaging of the brain during visual stimulation. Ultrasound Med Biol (2007) 33(5):681–90. 10.1016/j.ultrasmedbio.2006.11.008 17346872 PMC1995427

[58] Linninger AA Xenos M Sweetman B Ponkshe S Guo X Penn R. A mathematical model of blood, cerebrospinal fluid and brain dynamics. J Math Biol (2009) 59(6):729–59. 10.1007/s00285-009-0250-2 19219605

[59] Savolainen S Paljärvi L Vapalahti M. Prevalence of Alzheimer’s disease in patients investigated for presumed normal pressure hydrocephalus: a clinical and neuropathological study. Acta Neurochir (Wien) (1999) 141(8):849–53. 10.1007/s007010050386 10536721

[60] Bech RA Waldemar G Gjerris F Klinken L Juhler M. Shunting effects in patients with idiopathic normal pressure hydrocephalus; correlation with cerebral and leptomeningeal biopsy findings. Acta Neurochir (Wien) (1999) 141(6):633–9. 10.1007/s007010050353 10929729

[61] Golomb J Wisoff J Miller DC Boksay I Kluger A Weiner H Alzheimer’s disease comorbidity in normal pressure hydrocephalus: prevalence and shunt response. J Neurol Neurosurg Psychiatry (2000) 68(6):778–81. 10.1136/jnnp.68.6.778 10811706 PMC1736969

[62] Cabral D Beach TG Vedders L Sue LI Jacobson S Myers K Frequency of Alzheimer’s disease pathology at autopsy in patients with clinical normal pressure hydrocephalus. Alzheimers Dement (2011) 7(5):509–13. 10.1016/j.jalz.2010.12.008 21723206 PMC3166980

[63] Streitberger K-J Wiener E Hoffmann J Freimann FB Klatt D Braun J In vivo viscoelastic properties of the brain in normal pressure hydrocephalus. NMR Biomed (2011) 24(4):385–92. 10.1002/nbm.1602 20931563

[64] Silverberg GD Mayo M Saul T Rubenstein E McGuire D. Alzheimer’s disease, normal-pressure hydrocephalus, and senescent changes in CSF circulatory physiology: a hypothesis. Lancet Neurol (2003) 2(8):506–11. 10.1016/S1474-4422(03)00487-3 12878439

[65] Rubenstein E. Relationship of senescence of cerebrospinal fluid circulatory system to dementias of the aged. Lancet (1998) 351(9098):283–5. 10.1016/S0140-6736(97)09234-9 9457114

[66] Chakravarty A. Unifying concept for Alzheimer’s disease, vascular dementia and normal pressure hydrocephalus - a hypothesis. Med Hypotheses (2004) 63(5):827–33. 10.1016/j.mehy.2004.03.029 15488655

[67] De la Torre JC. Cerebral hemodynamics and vascular risk factors: setting the stage for Alzheimer’s disease. J Alzheimers (2012) 32(3):553–67. 10.3233/JAD-2012-120793 22842871

[68] Lee SJ. A possible pathogenesis for Alzheimer’s disease: craniomaxillofacial dysfunction leading to localized cerebrospinal fluid stasis. Med Hypotheses (2009) 72(2):199–210. 10.1016/j.mehy.2008.09.044 19026494

[69] O’Rourke MF Hashimoto J. Mechanical factors in arterial aging: a clinical perspective. J Am Coll Cardiol (2007) 50(1):1–13. 10.1016/j.jacc.2006.12.050 17601538

[70] Safar ME Nilsson PM Blacher J Mimran A. Pulse pressure, arterial stiffness, and end-organ damage. Curr Hypertens Rep (2012) 14(4):339–44. 10.1007/s11906-012-0272-9 22555981

[71] Hughes TM Kuller LH Barinas-Mitchell EJM Mackey RH McDade EM Klunk WE Pulse wave velocity is associated with β-amyloid deposition in the brains of very elderly adults. Neurology (2013) 81(19):1711–8. 10.1212/01.wnl.0000435301.64776.37 24132374 PMC3812104

[72] Zhong W Cruickshanks KJ Schubert CR Carlsson CM Chappell RJ Klein BEK Pulse wave velocity and cognitive function in older adults. Alzheimer Dis Assoc Disord (2013) 28:44–9. 10.1097/WAD.0b013e3182949f06 23632267 PMC3778043

[73] Hardy J Allsop D. Amyloid deposition as the central event in the aetiology of Alzheimer’s disease. Trends Pharmacol Sci (1991) 12(10):383–8. 10.1016/0165-6147(91)90609-V 1763432

[74] Breunig JJ Guillot-Sestier M-V Town T Brain injury, neuroinflammation and Alzheimer’s disease. Front Aging Neurosci (2013) 5:26 10.3389/fnagi.2013.00026 23874297 PMC3708131

[75] Klinge PM Samii A Niescken S Brinker T Silverberg GD. Brain amyloid accumulates in aged rats with kaolin-induced hydrocephalus. Neuroreport (2006) 17(6):657–60. 10.1097/00001756-200604240-00020 16603930

[76] Silverberg GD Miller MC Machan JT Johanson CE Caralopoulos IN Pascale CL Amyloid and tau accumulate in the brains of aged hydrocephalic rats. Brain Res (2010) 1317:286–96. 10.1016/j.brainres.2009.12.065 20045398

[77] Carnevale D Lembo G. “Alzheimer-like” pathology in a murine model of arterial hypertension. Biochem Soc Trans (2011) 39(4):939–44. 10.1042/BST0390939 21787327

[78] Schreiber S Drukarch B Garz C Niklass S Stanaszek L Kropf S Interplay between age, cerebral small vessel disease, parenchymal amyloid-β, and tau pathology: longitudinal studies in hypertensive stroke-prone rats. J Alzheimers Dis (2014) 42(Suppl 3):S205–15. 10.3233/JAD-132618 24825568

[79] Spires TL Hyman BT. Transgenic models of Alzheimer’s disease: learning from animals. NeuroRx (2005) 2(3):423–37. 10.1602/neurorx.2.3.423 16389306 PMC1144486

[80] LaFerla FM Green KN Animal models of Alzheimer disease. Cold Spring Harb Perspect Med (2012) 2(11) 423–37. 10.1101/cshperspect.a006320 PMC3543097 23002015

[81] Duyckaerts C Potier M-C Delatour B. Alzheimer disease models and human neuropathology: similarities and differences. Acta Neuropathol (Berl) (2008) 115(1):5–38. 10.1007/s00401-007-0312-8 18038275 PMC2100431

[82] Cohen RM Rezai-Zadeh K Weitz TM Rentsendorj A Gate D Spivak I A transgenic Alzheimer rat with plaques, tau pathology, behavioral impairment, oligomeric A? and frank neuronal loss. J Neurosci (2013) 33(15):6245–56. 10.1523/JNEUROSCI.3672-12.2013 23575824 PMC3720142

[83] Ojo J-O Mouzon B Greenberg MB Bachmeier C Mullan M Crawford F. Repetitive mild traumatic brain injury augments tau pathology and glial activation in aged htau mice. J Neuropathol Exp Neurol (2013) 72(2):137–51. 10.1097/NEN.0b013e3182814cdf 23334597

[84] Abrahamson EE Ikonomovic MD Ciallella JR Hope CE Paljug WR Isanski BA Caspase inhibition therapy abolishes brain trauma-induced increases in Abeta peptide: implications for clinical outcome. Exp Neurol (2006) 197(2):437–50. 10.1016/j.expneurol.2005.10.011 16300758

[85] Yu F Zhang Y Chuang D-M. Lithium reduces BACE1 overexpression, β amyloid accumulation, and spatial learning deficits in mice with traumatic brain injury. J Neurotrauma (2012) 29(13):2342–51. 10.1089/neu.2012.2449 22583494 PMC3430485

[86] Blasko I Beer R Bigl M Apelt J Franz G Rudzki D Experimental traumatic brain injury in rats stimulates the expression, production and activity of Alzheimer’s disease beta-secretase (BACE-1). J Neural Transm (2004) 111(4):523–36. 15057522 10.1007/s00702-003-0095-6

[87] Tran HT LaFerla FM Holtzman DM Brody DL. Controlled cortical impact traumatic brain injury in 3xTg-AD mice causes acute intra-axonal amyloid-β accumulation and independently accelerates the development of tau abnormalities. J Neurosci (2011) 31(26):9513–25. 10.1523/JNEUROSCI.0858-11.2011 21715616 PMC3146343

[88] Israelsson C Bengtsson H Kylberg A Kullander K Lewén A Hillered L Distinct cellular patterns of upregulated chemokine expression supporting a prominent inflammatory role in traumatic brain injury. J Neurotrauma (2008) 25(8):959–74. 10.1089/neu.2008.0562 18665806

[89] Smith DH Chen XH Nonaka M Trojanowski JQ Lee VM Saatman KE Accumulation of amyloid beta and tau and the formation of neurofilament inclusions following diffuse brain injury in the pig. J Neuropathol Exp Neurol (1999) 58(9):982–92. 10.1097/00005072-199909000-00008 10499440 PMC13479983

[90] Lewén A Li GL Nilsson P Olsson Y Hillered L. Traumatic brain injury in rat produces changes of beta-amyloid precursor protein immunoreactivity. Neuroreport (1995) 6(2):357–60. 10.1097/00001756-199501000-00032 7756628

[91] Yatsiv I Grigoriadis N Simeonidou C Stahel PF Schmidt OI Alexandrovitch AG Erythropoietin is neuroprotective, improves functional recovery, and reduces neuronal apoptosis and inflammation in a rodent model of experimental closed head injury. FASEB J (2005) 19(12):1701–3. 16099948 10.1096/fj.05-3907fje

[92] Raghupathi R Conti AC Graham DI Krajewski S Reed JC Grady MS Mild traumatic brain injury induces apoptotic cell death in the cortex that is preceded by decreases in cellular Bcl-2 immunoreactivity. Neuroscience (2002) 110(4):605–16. 10.1016/S0306-4522(01)00461-4 11934469

[93] Pierce JE Trojanowski JQ Graham DI Smith DH McIntosh TK. Immunohistochemical characterization of alterations in the distribution of amyloid precursor proteins and beta-amyloid peptide after experimental brain injury in the rat. J Neurosci (1996) 16(3):1083–90. 8558237 10.1523/JNEUROSCI.16-03-01083.1996 PMC6578806

[94] Bramlett HM Kraydieh S Green EJ Dietrich WD. Temporal and regional patterns of axonal damage following traumatic brain injury: a beta-amyloid precursor protein immunocytochemical study in rats. J Neuropathol Exp Neurol (1997) 56(10):1132–41. 10.1097/00005072-199710000-00007 9329457

[95] Hoshino S Tamaoka A Takahashi M Kobayashi S Furukawa T Oaki Y Emergence of immunoreactivities for phosphorylated tau and amyloid-beta protein in chronic stage of fluid percussion injury in rat brain. Neuroreport (1998) 9(8):1879–83. 10.1097/00001756-199806010-00039 9665619

[96] Iwata A Chen X-H McIntosh TK Browne KD Smith DH. Long-term accumulation of amyloid-beta in axons following brain trauma without persistent upregulation of amyloid precursor protein genes. J Neuropathol Exp Neurol (2002) 61(12):1056–68. 12484568 10.1093/jnen/61.12.1056

[97] Carbonell WS Grady MS. Regional and temporal characterization of neuronal, glial, and axonal response after traumatic brain injury in the mouse. Acta Neuropathol (1999) 98(4):396–406. 10.1007/s004010051100 10502046

[98] Hutchison JS Derrane RE Johnston DL Gendron N Barnes D Fliss H Neuronal apoptosis inhibitory protein expression after traumatic brain injury in the mouse. J Neurotrauma (2001) 18(12):1333–47. 10.1089/08977150152725632 11780864

[99] Yoshiyama Y Uryu K Higuchi M Longhi L Hoover R Fujimoto S Enhanced neurofibrillary tangle formation, cerebral atrophy, and cognitive deficits induced by repetitive mild brain injury in a transgenic tauopathy mouse model. J Neurotrauma (2005) 22(10):1134–41. 10.1089/neu.2005.22.1134 16238489

[100] Kurata T Lukic V Kozuki M Wada D Miyazaki K Morimoto N Telmisartan reduces progressive accumulation of cellular amyloid beta and phosphorylated tau with inflammatory responses in aged spontaneously hypertensive stroke resistant rat. J Stroke Cerebrovasc Dis (2014). 10.1016/j.jstrokecerebrovasdis.2014.05.023 25241340

[101] Nonaka Y Miyajima M Ogino I Nakajima M Arai H. Analysis of neuronal cell death in the cerebral cortex of H-Tx rats with compensated hydrocephalus. J Neurosurg Pediatr (2008) 1(1):68–74. 10.3171/PED-08/01/068 18352806

[102] Mori F Tanji K Yoshida Y Wakabayashi K. Thalamic retrograde degeneration in the congenitally hydrocephalic rat is attributable to apoptotic cell death. Neuropathol (2002) 22(3):186–93. 10.1046/j.1440-1789.2002.00445.x 12416558

[103] Del Bigio MR Zhang YW. Cell death, axonal damage, and cell birth in the immature rat brain following induction of hydrocephalus. Exp Neurol (1998) 154(1):157–69. 10.1006/exnr.1998.6922 9875277

[104] Aoyama Y Kinoshita Y Yokota A Hamada T. Neuronal damage in hydrocephalus and its restoration by shunt insertion in experimental hydrocephalus: a study involving the neurofilament-immunostaining method. J Neurosurg (2006) 104(5 Suppl):332–9. 16848091 10.3171/ped.2006.104.5.332

[105] Khan OH Enno TL Del Bigio MR. Brain damage in neonatal rats following kaolin induction of hydrocephalus. Exp Neurol (2006) 200(2):311–20. 10.1016/j.expneurol.2006.02.113 16624304

[106] Silverberg GD Miller MC Pascale CL Caralopoulos IN Agca Y Agca C Kaolin-induced chronic hydrocephalus accelerates amyloid deposition and vascular disease in transgenic rats expressing high levels of human APP. Fluids Barriers CNS (2015) 12(1):2. 10.1186/2045-8118-12-2 25685319 PMC4328504

[107] Levy Nogueira M Lafitte O Steyaert J-M Bakardjian H Dubois B Hampel H Mechanical stress related to brain atrophy in Alzheimer’s disease. Alzheimers Dement (2015). 10.1016/j.jalz.2015.03.005 26086185

[108] Murphy MC Curran GL Glaser KJ Rossman PJ Huston J III Poduslo JF Magnetic resonance elastography of the brain in a mouse model of Alzheimer’s disease: initial results. Magn Reson Imaging (2012) 30(4):535–9. 10.1016/j.mri.2011.12.019 22326238 PMC3433281

[109] Murphy MC Huston J III Jack CR Jr Glaser KJ Senjem ML Chen J Measuring the characteristic topography of brain stiffness with magnetic resonance elastography. PLoS One (2013) 8(12):e81668. 10.1371/journal.pone.0081668 24312570 PMC3847077

[110] Tyler WJ. The mechanobiology of brain function. Nat Rev Neurosci (2012) 13(12):867–78. 10.1038/nrn3383 23165263

[111] Manduca A Oliphant TE Dresner MA Mahowald JL Kruse SA Amromin E Magnetic resonance elastography: non-invasive mapping of tissue elasticity. Med Image Anal (2001) 5(4):237–54. 10.1016/S1361-8415(00)00039-6 11731304

[112] Murphy MC Huston J Jack CR Glaser KJ Manduca A Felmlee JP Decreased brain stiffness in Alzheimer’s disease determined by magnetic resonance elastography. J Magn Reson Imaging (2011) 34(3):494–8. 10.1002/jmri.22707 21751286 PMC3217096

